# Role of Microencapsulated Essential Oil and Pepper Resin in the Diet of Cows in the Third Lactation Phase on Immunological Pathways

**DOI:** 10.3390/vetsci12040344

**Published:** 2025-04-08

**Authors:** Karoline Wagner Leal, Marta Lizandra do Rego Leal, Gabriel S. Klein, Andrei Lucas R. Brunetto, Guilherme Luiz Deolindo, Camila Eduarda Justen, Matheus Dellaméa Baldissera, Tainara L. Santos, Daniela Zanini, Rafael C. de Araujo, Aleksandro Schafer da Silva

**Affiliations:** 1 Graduate Program in Veterinary Medicine, Federal University of Santa Maria, Santa Maria 97105-900, RS, Brazil; 2 Department of Large Animal Clinic, Federal University of Santa Maria, Santa Maria 97105-900, RS, Brazil; marta.leal@ufsm.br; 3 Department of Animal Science, State University of Santa Catarina, Chapecó 89815-630, SC, Brazil; gabrielsasseti@gmail.com (G.S.K.); andrei.brunetto@edu.udesc.br (A.L.R.B.); guilherme.ld073@edu.udesc.br (G.L.D.); 07719519906@edu.udesc.br (T.L.S.); 4 Center of Health Science, Universidade Franscicana, Santa Maria 97010-032, RS, Brazil; camila.eduarda.justen@ufn.br (C.E.J.); matheus.dellamea@ufn.edu.br (M.D.B.); 5 Department of Health Science, Universidade da Fronteira Sul, Chapecó 89813-140, SC, Brazil; dz_daniela@yahoo.com.br; 6 GRASP Ind. & Com. Ltda, Department of Research and Development—Ruminant Division, Curitiba 81260-000, PR, Brazil; rafael@grasp.ind.br

**Keywords:** cattle, nutraceuticals, cell signaling, anti-inflammatory, homeostasis

## Abstract

The objective was to determine whether dairy cows may activate traditional and alternative inflammatory pathways by consuming a combination of a phytogenic diet (essential oil and pepper resin). Twenty pregnant Jersey cows in the final (third) lactation phase (260 days in milk) were divided into two groups: control, with no additive consumption, and test, with the addition of the phytogenic to the concentrate portion of the diet (150 mg/day/kg dry matter). Blood samples were collected on experimental days 1, 7, 14, 21, 28, 35, and 42 by coccygeal vein puncture to assess the complete blood count, serum biochemistry of levels of total protein, albumin, and globulin, as well as carbohydrate metabolism (glucose), lipid metabolism (cholesterol and triglycerides), protein metabolism (urea), activities of hepatic enzymes (gamma-glutamyl transferase (GGT) and aspartate aminotransferase (AST)), cytokine levels (interleukins IL-1β, IL-6, and IL-10), antioxidant response [thiobarbituric acid reactive substances (TBARS), reactive oxygen species (ROS), total thiol (PSH), and non-protein thiol (NPSH), and glutathione S(GST)], cholinergic system [total cholinesterase (ChE) and acetylcholinesterase (AChE)], purinergic signaling [NTPDase, 5′ectonucleotidase and adenosine deaminase (ADA)], and energetic metabolism enzymes [creatine kinase (CK), pyruvate kinase (PK), and adenylate kinase (AK)]. Productive performance was assessed through feed intake and milk production. The results revealed that the use of phytogenic compounds significantly influenced the cholinergic system and purinergic signaling associated with immunology. The reduction in cholinesterase (ChE) activity and the increase in acetylcholinesterase (AChE) activity in lymphocytes suggest the modulation of the cholinergic system, enhancing the immune response. Furthermore, the elevated activity of adenosine deaminase (ADA) in lymphocytes and platelets, together with increased ATP and ADP hydrolysis in platelets, indicates the beneficial regulation of purinergic signaling, potentially contributing to inflammatory modulation. These effects were accompanied by a lower production of pro-inflammatory cytokines (IL-1β and IL-6) and a higher production of IL-10, reinforcing an anti-inflammatory profile. The reduced leukocyte and lymphocyte counts may reflect a lower inflammatory demand, while the increased levels of NPSH and GST antioxidants suggest cellular protection. Despite these physiological changes, productive performance and milk quality remained unaffected. In summary and practical terms, including this additive in the cows’ diet benefits the cow’s health in the final third of gestation when the animal already has a reduced immune response due to advanced gestation.

## Simple Summary

Phytogenics based on carvacrol, eugenol, cinnamaldehyde, and capsaicin improved animal health due to their anti-inflammatory, antioxidant, and immunomodulatory activities. The additive may be a promising alternative to reducing metabolic stress during lactation. Indications that phytogenics influence immune cholinergic and purinergic signaling pathways were observed, although their effects on energetic metabolism are related to ATP production. The metabolic effects observed in the overall health of Jersey cows fed phytogenics did not negatively impact productive performance.

## 1. Introduction

As health promoters, essential oils can enhance feed efficiency by reducing metabolic and digestive disorders in ruminants. Their potential to improve animal health, immunity, and productivity has been scientifically documented, including during the third phase of lactation, a critical period for animal health [1,2,3]. This eco-efficient and natural approach to animal nutrition aligns with the sustainable development goals of world dairy farming. Inflammation is a fundamental physiological process that plays a crucial role in immune defense. However, excessive or chronic inflammation can compromise animal health and productivity, particularly in dairy cows during critical physiological stages, as the third phase of lactation [4]. Understanding how to modulate inflammation effectively is essential for optimizing both welfare and production efficiency.

The addition of a blend of essential oils (carvacrol, eugenol, cinnamaldehyde, and capsaicin) to the diet of Holstein cows resulted in a significant improvement in feed efficiency, as dry matter intake was reduced and milk production increased in the treated group [5]. Recent data from a pilot study demonstrated that the consumption of phytogenics based on carvacrol, eugenol, cinnamaldehyde, and capsaicin also stimulates the anti-inflammatory response, antioxidant capacity, and humoral immune response in Jersey cows [6]. While the effects of essential oils on traditional inflammatory biomarkers are known, recent research suggests that alternative inflammatory pathways may also play a critical role in immune modulation. Nevertheless, we hypothesized that other pathways would participate in this modulation of inflammation, including the cholinergic anti-inflammatory and purinergic immunomodulatory pathways during a critical period of life, as the third phase of lactation.

In this context, purinergic signaling is a ubiquitous cellular communication system composed of extracellular signaling molecules represented by nucleotides (ATP, ADP, and AMP) and their derived nucleoside adenosine, controlled by the enzymatic action of E-NTPDases, 5′-Nucleotidases, and adenosine deaminases. These biomolecules regulate various physiological and pathological processes with anti-inflammatory and immunomodulatory actions [7,8]. It has also been well documented that in addition to neuronal cholinergic systems, non-neuronal cholinergic systems exist, which simultaneously play a role in the inflammatory response [9,10,11]. Acetylcholine (ACh), a neurotransmitter, is also involved in cell-to-cell communication and controls cellular functions, such as reducing the proliferation of pro-inflammatory cytokines [12,13]. Given the growing interest in sustainable and antibiotic-free strategies for disease prevention in livestock, investigating the impact of essential oils on these alternatives signaling pathways could provide new insights into non-pharmacological methods for managing inflammation. Therefore, it is believed that essential oils could also influence these cellular signaling pathways, offering a potentially beneficial approach for modulating inflammation and promoting health in dairy cows during the third lactation phase, because in this phase, the animals have a reduction in their immunological system [14].

Hypothetically, activating purinergic and cholinergic signaling pathways in immune system cells can regulate cytokine production and promote an effective anti-inflammatory and immunomodulatory response. This effect could have significant implications for developing new therapies for inflammatory and immune disorders, as modulating the activity of these cellular and extracellular processes may enhance the body’s ability to maintain homeostasis. Thus, the innovation of the present study is identifying the involvement of cholinergic and purinergic pathways during the third lactation phase of cows, a critical period associated with several metabolic, hormonal, and immunological alterations [4], to understand how these pathways contribute to immunological chances in this period. Moreover, another important innovation of the study is to evaluate the possible modulative effects of additives based on natural products capable of modulating the cholinergic and purinergic pathways, improving immune response. By identifying potential mechanisms through which essential oils influence these pathways, this study aims to contribute to the development of innovative nutritional strategies that optimize immune function while improving animal performance. Therefore, the objective was to evaluate whether traditional and alternative inflammatory pathways may be activated by dairy cows’ intake of a combination of phytogenic compounds in their diet (essential oil and pepper resin).

## 2. Materials and Methods

### 2.1. Additive

The commercial additive based on microencapsulated EO of oregano and cinnamon and pepper resin (Activo Premium^®^; GRASP, Curitiba, PR, Brazil), an additive already used in a previous study in the peak production phase [6]: carvacrol (terpenoids), eugenol, cinnamaldehyde (phenylpropanoids), and red pepper resin oil (alkaloids); being that active components represent 15% of the product.

### 2.2. Experimental Condition

The study was carried out at the UDESC Experimental Farm, located in the western region of Santa Catarina (humid subtropical and mild mesothermal climate). The experiment used 20 pregnant Jersey cows, 3 years old, at the end of lactation (260 ± 53 days in lactation). All animals were apparently healthy. The cows were housed in a compost barn system and were milked (05:30 and 15:30) using a guided robotic milking system, model AMI 5550 (DeLaval VMSTM, Switzerland, Sweden). The milking system was only turned off at night, due to problems with the power grid on the farm, which impaired its operation.

### 2.3. Experimental Design and Feed

Twenty lactating primiparous cows were used in a completely randomized design, divided into two groups of ten cows each. The distribution of the animals considered the projected milk production for 305 days (260 ± 53 days in lactation; 20 ± 2 L of milk per day), as well as the body condition score (BCS). The first fourteen days were defined as the adaptation period, and the experimental period was defined between days 15 and 42, when data and samples were collected. The total mixed ration (TMR) was calculated based on the nutritional requirements of dairy cattle to meet the production of 20 L of milk per day [15], which corresponded to a net energy of 1.59 Mcal/kg of dry matter (24.6 Mcal/cow/day). The cows received the diet three times a day in individual feeders (06:00, 11:00, and 17:00), with water ad libitum during the feeding period and also when they were resting on the wood shavings bed. Feed additive was added during the concentrate production in a proportion that guaranteed the consumption of 150 mg/kg of dry matter in the treated group. The control group consumed the same TMR but without the additive. The chemical composition of the feeds is in Table 1.

### 2.4. Sample Collection

On days 1, 7, 14, 21, 28, 35, and 42, blood was collected from the cows by coccygeal venipuncture and using a vacuum collection system (BD Vacutainer^®^, Franklin Lakes, NJ, USA). Tubes with anticoagulant (EDTA) were used to perform hematological analyses as well as tubes containing clot activator; this material was used to obtain serum. After collection, the blood was placed in a thermal box with recyclable ice (±10 °C) during transportation to the laboratory. All blood samples collected were used to measure blood count and serum biochemistry; only samples collected on days 14, 28, and 42 of the experiment were used to assess oxidative status and immunological biomarkers.

### 2.5. Hemogram

Using a three-part hematology analyzer (Equip^®^ Vet 3000, Itatiba, SP, Brazil), erythrocytes and leukocytes were measured, as well as hemoglobin concentration and hematocrit percentage. The automatic equipment provides the results instantly in Excel spreadsheets, which allows for rapid descriptive analysis and, if necessary, repeating the test using the same sample.

### 2.6. Serum Biochemistry

Blood serum was obtained by centrifugation with a clot activator in a centrifuge for QUIMIS^®^ tubes (Diadema, SP, Brazil) for 10 min with a force of 7871 g and stored in Eppendorf tubes at −20 °C for subsequent biochemical analyses. Total protein (TP), albumin, glucose, cholesterol, triglycerides, and urea levels, as well as gamma-glutamyl transferase (GGT), aspartate aminotransferase (AST), and cholinesterase (ChE) enzyme activities, were measured in serum. Globulin levels were calculated [Globulin = TP − albumin]. We used an automatic analyzer (Zybio EXC-200, Shenzhen, Guangdong Province, China) with commercial Analisa^®^ kits (Gold Analisa Diagnostica Ltda., Belo Horizonte, MG, Brazil), according to their respective methodologies to determine the biochemical profile listed above. Beta-hydroxybutyrate levels were determined with a portable digital device, Free Style Optium Neo^®^ (Abbott^®^, Chicago, IL, USA), from blood β-ketone test strips (Abbott^®^, Chicago, IL, USA).

### 2.7. Cytokines—Interleukin 1β, Interleukin 6, and Interleukin 10

Interleukins (IL-1β, IL-6, and IL-10) were quantified using enzyme-linked immunosorbent assays with commercial kits, and according to the manufacturer’s instructions (R&D Systems, Minneapolis, MN, USA). The color intensity was measured using a micro-ELISA reader to determine the cytokine concentrations.

### 2.8. TBARS Levels

TBARS levels were measured using malondialdehyde (MDA) as the primary product. The technique is based on the reaction of MDA with thiobarbituric acid (TBA), resulting in a color change (pink), which is quantified by spectrophotometry with absorbance (532 nm, after 45 min incubation at 37 °C) [16,17]. According to technical instructions, 20 µL of samples were mixed with 55 µL of distilled water, 100 µL of orthophosphoric acid (0.2 M), and 25 µL of TBA (0.1 M). The spectrophotometric reading was performed after 45 min of incubation at 37 °C. The results were expressed in nM MDA/mL of serum.

### 2.9. Reactive Oxygen Species (ROS)

Fluorimetric techniques were used for ROS quantification [18,19,20]. ROS production is determined indirectly by the estimation of 2′,7′-dichlorofluorescein diacetate (DCFH-DA), which is oxidized to dichlorofluorescein (DCF), a fluorescent molecule. A volume of 10 µL of serum was mixed with 10 µL of DCFH-DA (7 μM) and 240 µL of PBS, and after 30 min of incubation at 37 °C, the final oxidation product, DCF, was measured (525 nm and 488 nm) (Thermo Scientific™ Varioskan™ LUX, Waltham, MA, USA). The results were expressed in units of DCFH per milligram of protein.

### 2.10. Levels of Total Thiol (PSH) and Non-Protein Thiol (NPSH)

Total and non-protein thiols were measured using the method described by Ellman [21], with adjustments for a 96-well plate format. A volume of 30 µL of serum was added to a 96-well plate, as well as 200 µL of potassium phosphate buffer (PPB) and 20 µL of 5,5′-dithiobis(2-nitrobenzoic acid) (DTNB). The material was first deproteinized to 10% with an equal volume of trichloroacetic acid (TCA) to measure the non-protein thiol fraction, and the remaining supernatant was used. Then, 40 µL of the supernatant was combined with 260 µL of PPB and 15 µL of DTNB, with immediate reading. An immediate reading was then performed on a spectrophotometer. The results were compared to a cysteine standard curve and expressed in micromolarity (µM).

### 2.11. Glutathione S-Transferase (GST) Activity

Glutathione S-transferase (GST) activity was determined based on Habig et al. [22]. The technique used was dependent on the ability of GST to catalyze the conjugation of reduced glutathione (GSH) with 1-chloro-2,4-dinitrobenzene (CDNB). GST activity was measured as the rate of dinitrophenyl-S-glutathione formation at 340 nm in medium containing 50 mM of potassium phosphate at pH 6.5, 1 mM of GSH, 1 mM of CDNB as substrate, and tissue supernatants (approximately 0.045 mg of protein). Results showed in U GST/mg protein.

### 2.12. Purinergic Signaling: Separation of Lymphocytes and Platelets

The isolation of peripheral blood lymphocytes was performed using Ficoll- Histopaque™ plus (Sigma-Aldrich Brasil Ltda, Barueri, SP, Brazil) to form density gradients according to the methodology described by Bøyum [23]. The separation of platelets was carried out according to the method described by Pilla et al. [24]. Cell viability was estimated by lactate dehydrogenase release before and after incubation at 37 °C using the kinetic method of the Labquest apparatus (Gold Analisa Diagnostica Ltda., Belo Horizonte, MG, Brazil).

Quantification of protein levels using bovine serum albumin as a standard followed the Bradford method [25]; the proteins were adjusted in each assay (mg/mL).

Adenosine deaminase (ADA) activity in lymphocytes was measured based on the ammonia produced when this enzyme acts on adenosine [26]. To begin the analysis, the substrate (adenosine) was added to a final concentration of 21 mmol/L and incubations were carried out for 1 h at 37 °C. The reaction was stopped by adding 106 mmol/L/0.16 mmol/L of phenolnitroprusside/mL solution. Then the solution was mixed with 125 mmol/L/11 mmol/L sodium hypochlorite and shaken. Ammonium sulfate (75 μmol/L) was used as a standard for ammonia, which had its levels determined from the absorption of indophenol at 620 nm. Results were presented at U/mg of protein.

Acetylcholinesterase (AChE) activity in lymphocytes was determined using the spectrophotometric method [27]. All samples had protein levels adjusted to 0.1–0.2 mg/mL. Then, 0.2 mL volume of lymphocytes were added to a solution containing 1.0 mM acetylthiocholine, 0.1 mM 2-nitrobenzoic acid, and 0.1 mM phosphate buffer at pH 8.0. Before and after incubation for 30 min at 27 °C, absorbance was read on a spectrophotometer at 412 nm. Enzyme activity was expressed as μmol of acetylthiocholine iodide (AcSCh) per hour per mg of protein.

According to Lunkes et al. [28] and Pilla et al. [24], to determine ATP/ADP hydrolysis, a reaction was designed containing 5 mM CaCl_2_, 100 mM NaCl, 5 mM KCl, 6 mM glucose, and 50 mM Tris-HCl buffer, pH 7.4. To determine AMP hydrolysis, we used 10 mM MgCl_2_ instead of CaCl_2_ compared to ATP/ADP hydrolysis. Protein normalization was performed in saline solution, then 20 μL of platelets were added to the reaction system and preincubated at 37 °C for 10 min adding the substrates ATP and ADP to determine NTPDase activity and AMP for 5′nucleotidase activity in an incubation system at 37 °C for 70 min. Reactions were stopped when 150 μL of 10% TCA was added. From each sample, 30 µL were transferred to a new plate, 300 µL of malachite green as a colorimetric reagent were added, and the inorganic phosphate released was measured at 630 nm in a spectrophotometer. A standard curve was made with KH_2_PO_4_, and the results were expressed in nmol of Pi released/min/mg of protein.

### 2.13. Enzymatic Energetic Metabolism

CK activity was determined in the reaction mixture containing the following final concentrations: 65 mM Tris-HCl buffer, pH 7.5, 7 mM PCr, 9 mM MgSO_4_, and 20 μL of sample. After 10 min of preincubation at 37 °C, the reaction was initiated by the addition of 0.3 μmol of ADP and stopped after 10 min by the addition of 1 μmol of hydroxymercuribenzoic acid. Then, the creatine concentration was obtained by the colorimetric method [29]; readings at 540 nm and results were expressed as U/L of creatine formed per minute per mg of protein. AK activity was verified with an enzymatic assay using hexokinase (HK) and glucose 6-phosphate dehydrogenase (G6PD) [30]. The reaction was initiated by the addition of 2 mM ADP, and the reduction of NADP+ was measured at 340 nm for 3 min in a spectrophotometer; the activity was expressed as mU/mL of ATP formed per min per mg of protein. PK activity was measured using the methodology fully described by Leong et al. [31], and the results were expressed as U/L of pyruvate formed per min per mg of protein.

### 2.14. Nutrient Analysis

TMR samples were predried in a forced ventilation oven (55 °C for 72 h), and then the material was weighed again to determine the partial dry matter content. This material underwent a grinding process in a Wiley mill (Marconi, model: MA340), with a 1 mm mesh sieve. Subsequently, the predried samples were placed in an oven (105 °C for 24 h), a methodology that allowed verifying the dry matter (DM) of the sample. To determine the ash, a muffle furnace at 600 °C was used [32]. The nitrogen content was measured by the micro-Kjeldahl method (Method 984.13); information was used to estimate the crude protein content (Method 976.05; CP = N × 6.25). The percentage of neutral detergent fiber (NDF) was measured by placing the ground TMR samples in polyester bags [33] and then exposing them to a neutral detergent solution in an autoclave at 110 °C for 40 min. [34]. We included in the reaction the heat-stable alpha-amylase: activity = 17,400 Liquefon Units/mL (FAA, ANKOM Technology, Macedon, NY, USA) [29]. The acid detergent fiber (ADF) ratio was measured according to method 973.18 [26]. The percentage of ether extract in the TMR was measured using a near-infrared reflectance spectrometer, model Spectra Star 2600 XT series of Near-Infrared Analyzers (Unity Scientific^®^, Ferntree Gully Rd, Knoxfield, Australia).

### 2.15. Productive Performance

Dairy cows were weighed with a digital scale on the first day to adjust the diet provided to the cows daily. Every time the cow passed the robot, the BCS was assessed daily using a high-resolution digital camera attached to the factory-installed robotic milking system. The robot’s software transformed these images into BCS numbers from 1 (low) to 5 (high).

The robotic milking system quantified daily milk production, individually for each papilla and per milking per day, and it averaged over seven days; these data were extracted into Excel spreadsheets. Somatic cell counts (SCCs) were determined automatically by the system attached to the daily robotic milking system, and this quantification was performed every time the cow was milked that day; at the end, the average SCC was calculated per day. During milking, up to 2 kg/day of pelleted feed (Cooper Alfa, Chapecó, Brazil) was provided to the cows automatically but recorded by the robot.

The study lasted 42 days, during which the feed supply (silage, concentrate, hay) was weighed individually, then mixed manually and offered to the cow in her feeder. Once a day, the leftovers were weighed and discarded. The amount supplied and the leftovers determined the food intake in natural matter. Then, the percentage of DM in the RMR was measured, which made it possible to calculate the food consumption in DM. The formula determined feed efficiency: milk production in kilograms/feed intake in DM per day in kilograms.

The milk production corrected for 4% fat (FCM) was calculated as follows: FCM = 0.4 × Milk production + 15 × Fat production, according to NRC.

### 2.16. Milk Analysis

Using the Delaval collection system (VMSTM Series) coupled to robotic milking, milk was collected on days 1, 14, 28, and 42. This material was used to measure the centesimal composition of the milk in a laboratory accredited by MAPA in Brazil, using the Fourier transform mid-infrared spectrometry methodology to determine the contents of total protein, lactose, fat, total solids, and urea nitrogen of the milk, expressed as percentages (Brazilian standard: ISO 9622/IDF 141:2013). Using flow cytometry following ISO 13366-2/IDF 148-2:2006, the somatic cell count in the milk was determined.

### 2.17. Statistical Analyses

Data were evaluated using the SAS ‘MIXED procedure’ (SAS Inst. Inc., Cary, NC, USA; version 9.4), with Satterthwaite approximation to determine the denominator degrees of freedom for the fixed effects test. Milk yield, TMR intake, and production efficiency were tested for treatment fixed effects using animal (treatment) as random effects. Milk yield and all whole blood and ruminal fluid results were analyzed as repeated measures; where for treatment fixed effects and treatment × day, using animal (treatment) as random effects. All data obtained from day 1 were included as an independent covariate. Means were tested by t-test, and results were presented as mean and SEM. Because the test allows, we considered significance when *p* ≤ 0.05, as well as a trend if *p* > 0.05 and ≤ 0.10.

## 3. Results

### 3.1. Blood Count and Serum Biochemistry

The blood count and serum biochemistry results are in Table 2. A treatment versus day interaction (*p* ≤ 0.05) was observed for leukocytes and lymphocytes. A significantly lower total leukocyte count was observed on days 7, 14, and 28 in the Phytogenic group, and lymphocyte count was significantly lower in the Phytogenic group compared to the control on days 7, 14, and 35. Urea concentration significantly decreased on day 28 in the Phytogenic group (*p* ≤ 0.05). AST activity was lower in the cows that intake the additive (*p* ≤ 0.02) on days 28 and 35. Cholinesterase activity was significantly lower in the Phytogenic group on days 28, 35, and 42 (*p* ≤ 0.01). There was no effect of treatment and treatment × day interaction for several granulocytes, monocytes, and platelets, hemoglobin concentration, and hematocrit percentage, as well as for total protein, albumin, cholesterol, glucose, triglycerides, GGT, and BHB (*p* > 0.05).

### 3.2. Levels of Cytokines and Oxidative Status

For cytokines, there was a treatment effect and treatment × day interaction (Table 3). Interleukin 1β and interleukin 6 levels were significantly lower in the Phytogenic group on days 14, 28, and 42 (*p* ≤ 0.01—Table 3). On the other hand, interleukin 10 levels were significantly higher on days 14, 28, and 42 in the Phytogenic group.

The TBARS levels were significantly lower in the Phytogenic group compared to the control (*p* ≤ 0.05—Table 3). There was a treatment × day interaction for ROS, NPSH, and GST. ROS quantification was significantly lower in the cows fed with phytogenics on days 28 and 42 (*p* < 0.01). NPSH levels were significantly higher in the Phytogenic group on days 14, 28, and 42 (*p* ≤ 0.01). GST activity was higher in the Phytogenic group only on day 42 (*p* ≤ 0.01).

### 3.3. Purinergic Signaling

**Figure 1:**
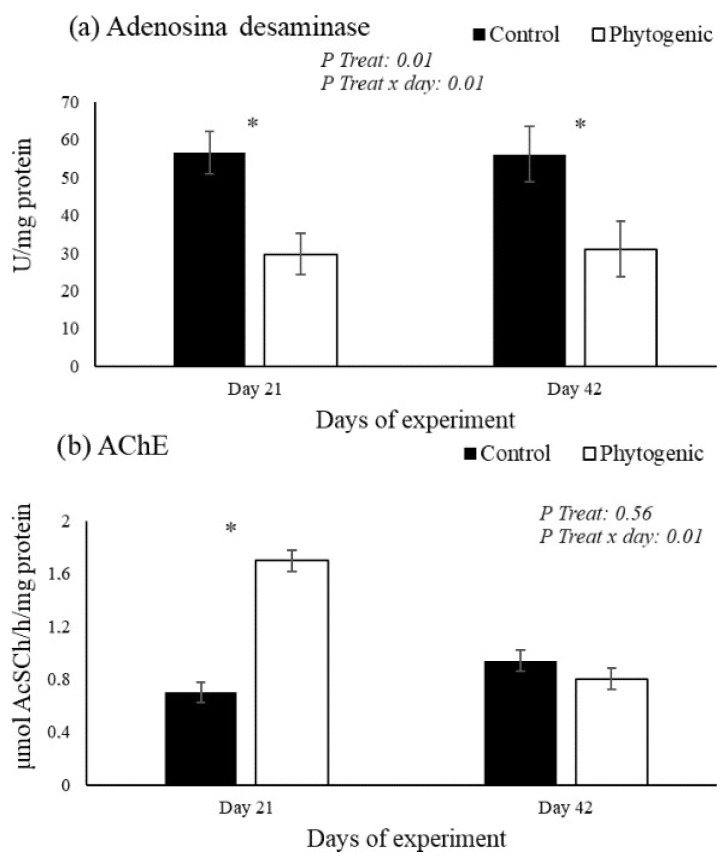
Activity of adenosine deaminase (**a**: ADA) and acetylcholinesterase (**b**: AChE) in lymphocytes from Jersey cows fed with the phytogenic. * Difference between of groups.

The results in lymphocytes are shown in Figure 1. The enzymatic activity of ADA in lymphocytes was lower in the Phytogenic group on days 21 and 42 compared to the control, with treatment effects (*p* ≤ 0.01) and treatment versus day interaction (*p* ≤ 0.01). The activity of AChE in lymphocytes was higher in the cows fed with the phytogenic on day 21 (*p* ≤ 0.01) compared to the control.

**Figure 2:**
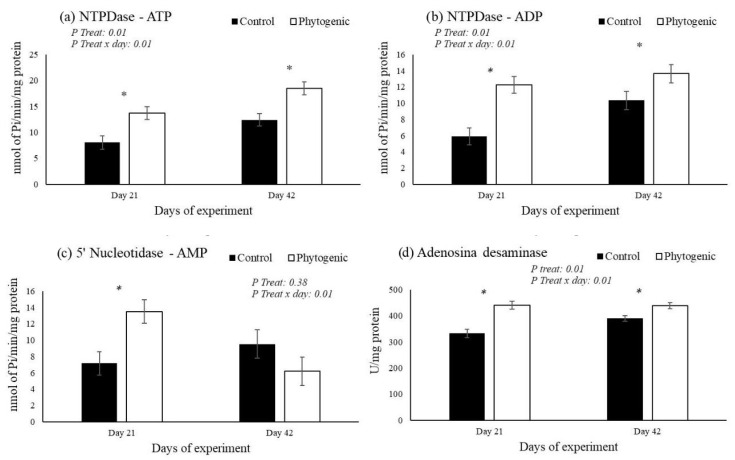
Activity of NTPDase ((**a**) ATP and (**b**) ADP substrates, 5′nucleotidase (**c**) AMP substrate, and adenosine deaminase (**d**) adenosine substrate) in platelets from cows fed with of phytogenics. * Difference between of groups.

The enzymatic activity of NTPDases (substrate ATP and ADP) in platelets was significantly higher in the Phytogenic group on days 21 and 42, with treatment effects (*p* ≤ 0.01) and treatment × day interaction (*p* ≤ 0.01). The enzymatic activity of 5′nucleotidase (substrate AMP) in platelets was higher in the Phytogenic group only on day 21 (*p* ≤ 0.01). The activity of ADA (substrate adenosine) in platelets was significantly higher in the Phytogenic group on days 21 and 42, with treatment effects (*p* ≤ 0.01) and treatment × day interaction (*p* ≤ 0.01). The results are shown in Figure 2.

### 3.4. Energetic Metabolism

**Figure 3:**
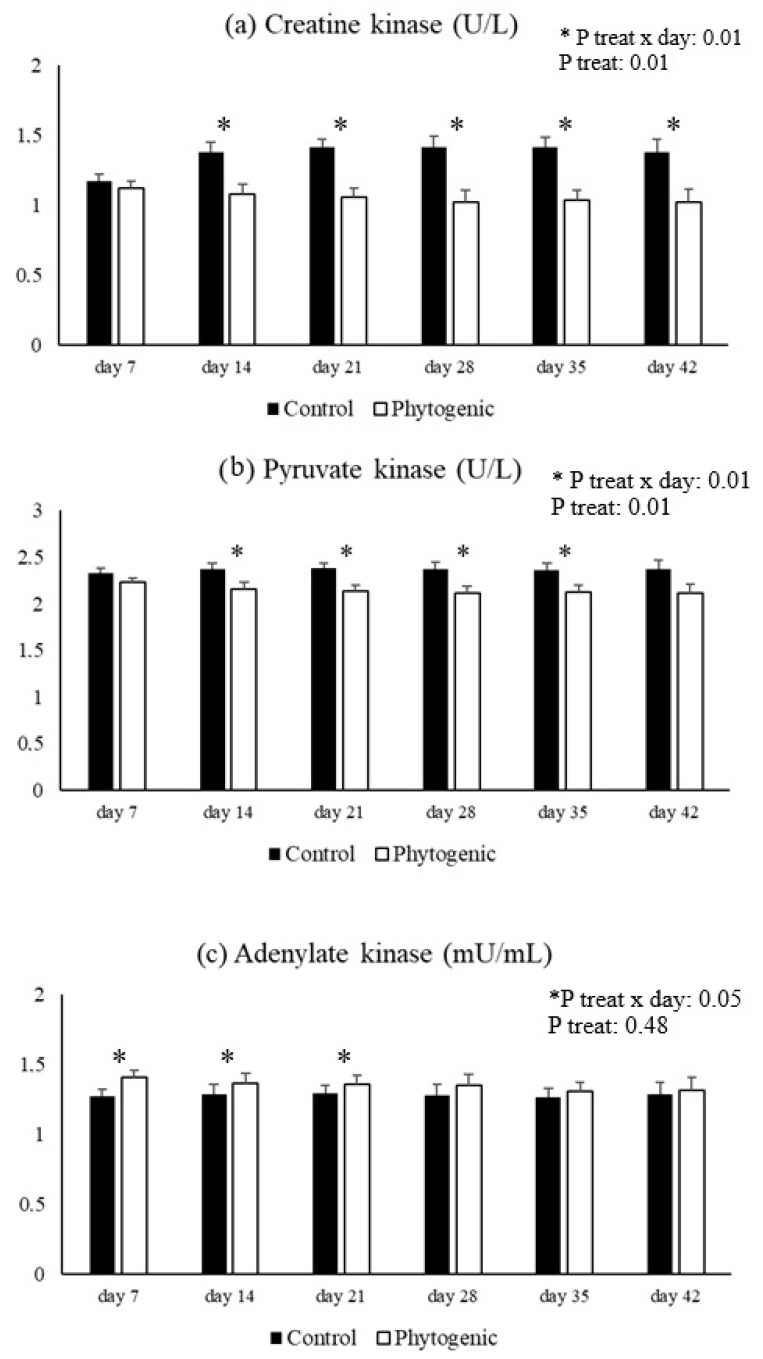
Enzymatic activity of creatine kinase (**a**), pyruvate kinase (**b**), and adenylate kinase (**c**) from Jersey cows fed with the phytogenic.

A treatment effect (*p* ≤ 0.01) and treatment × day interaction (*p* ≤ 0.01) for CK and PK activities. The enzymatic activity of CK was significantly lower in the Phytogenic group on days 14, 21, 28, 35, and 42, and PK activity was significantly lower in the cows fed with additives on days 14, 21, 28, and 35. The enzymatic activity of AK was significantly higher in the Phytogenic group on days 7, 14, and 21 (*p* ≤ 0.05) compared to the control (Figure 3).

### 3.5. Performance

The feed additive did not affect feed intake (14.12 kg of DM/day), milk production (15.42 L/day), feed efficiency, or BCS. There were no significant differences between groups in the fat, protein, lactose, total solids content, or SCC yields. The data are presented in Table 4.

## 4. Discussion

The health status assessment of the dairy cow must consider the general clinical context, lactation stage, diet, management, and environment. Analysis of blood count and biochemical profile are essential diagnostic methods for monitoring the general health status of animals [35]. Reference values for total leukocyte count in cattle generally range between 4000 and 12,000 leukocytes per µL of blood; our results are per the literary standard [36]. The lymphocyte count represents typically about 60% to 80% of the total leukocyte count [37]. Thus, in our study, we associated the reduction in the total leukocyte count with a decrease in the lymphocyte count. Essential oils can influence fluctuations in lymphocyte counts, but this mechanism of action is unclear. Hypothetically, it is believed that the antimicrobial effect exerted by essential oils reduces the infectious challenge; therefore, energy redirection occurs [38,39]. We suggest a remodeling of the inflammatory response due to the anti-inflammatory effect of the phytogenic based on carvacrol, eugenol, cinnamaldehyde, and capsaicin [40,41,42,43]. This result aligns with studies that used microencapsulated essential oils in ruminants [38,44]. Recent findings from a pilot study suggest anti-inflammatory action, with a reduction in leukocytes and lymphocytes in Jersey cows at the beginning of the lactation period (DEL 21 ± 1). In this study, we observed the behavior of these cells despite differences in the advancement of the lactation phase [6]. The anti-inflammatory potential of phytogenics may be an exciting alternative for lactating dairy cows as a therapeutic option for inflammatory disorders [45].

Recent findings indicate that the use of phytogenics may be related to the stimulation of the humoral immune response [44]; it has also been reported that the use of phytogenics in dairy cows increases the serum concentration of total proteins, globulins, with effects on the production of immunoglobulin A and heavy chain immunoglobulin [6]. Furthermore, although the decrease in urea nitrogen in our study was subtle, it may be due to the ability of essential oils to reduce the proteolysis and deamination of amino acids in the rumen, which leads to lower ammonia production [2]. This is beneficial because high ammonia levels in the rumen can harm the animal and result in nitrogen losses, a valuable protein synthesis resource.

These findings suggest lower protein metabolism and catabolism [46]. Although serum urea levels are often used to assess kidney function, when associated with liver enzyme activity, they can be helpful in the differential diagnosis of liver health. AST signals liver cell damage; however, it also plays a role in amino acid metabolism and is a critical intermediary molecule in cellular energy production [47]. It has been reported that phytogenics based on carvacrol [48], eugenol [49], cinnamaldehyde [50], and capsaicin [51] presented hepatoprotective activity; in our study, it is evident that the use of combined phytogenics presents synergy for this biological activity. Research has shown that advanced protein synthesis can be a hepatoprotective mechanism, which is a way for the body to contribute to accelerating the process of regeneration and production of liver cells [50]. This mechanism is explained by its negative regulation in oxidative and inflammatory processes, as well as its power to attenuate mitochondrial dysfunction and apoptosis [51].

Interleukins are signaling molecules involved in the modulation of the immune response, regulated by positive and negative feedback mechanisms to prevent excessive or insufficient immune responses [37]. Carvacrol inhibits the enzymatic activation of cyclooxygenase and causes anti-inflammatory effects by reducing the production of inflammatory mediators, such as IL-1β and proteinoids, possibly through the induction of the release of IL-10 responsible for suppressing excessive immune responses [41]. In our study, the production of pro-inflammatory cytokines (IL-1β and IL-6) decreased significantly, while IL-10 levels increased, suggesting synergy between phytogenic and proven anti-inflammatory action.

Treatment with the Phytogenic affected ROS and TBARS serum levels, i.e., our treatment reduced free radical formation and lipid damage. Phenolic compounds present in the structure of essential oils play important roles in the decomposition of peroxides and neutralization of free radicals due to their high reactivity with peroxyl radicals, which are eliminated by the formal transfer of hydrogen atoms, mediated by the redox property [52,53]. The cinnamaldehyde, the main component of cinnamon, can be linked to protective effects against ROS formation and consequently serum lipid damage, since it is a potent antioxidant that prevents formation of reactive species, preventing damage to proteins, lipids and nucleic acids [43,50].

NPSH, such as GST, contains a free thiol (-SH) group responsible for their antioxidant capacity. GST acts in cellular defense against oxidative stress and in the organism’s detoxification, both considered positive markers of redox balance [54]. Therefore, the increase in NPSH and GST observed in animals treated with phytogenics was expected, considering that the same additive was used in Jersey cows and presented similar behavior: lipid peroxidation, and carboline protein levels were reduced, and the enzymatic activity of GST and glutathione peroxidase was increased [6]. In general, it represents the redox balance essential for maintaining homeostasis, possibly due to the low consumption or reduced degradation of endogenous antioxidants by the antioxidant action of the phytogenic [49,55]. Moreover, this effect can be also attributed to cinnamaldehyde, since it has been studied for its effects on various detoxifying enzymes, including GST. The GST is crucial in cellular detoxification, helping to conjugate toxic substances with glutathione, rendering them more water-soluble for excretion [43], contributing to improvement of antioxidant defense system during the third phase of lactation. Finally, the protective effect on the antioxidant system can be also attributed to carvacrol, the main component of oregano essential oil [48]. Its antioxidant mechanism is primarily based on its chemical structure, which allows it to interact with ROS and other free radicals, thereby preventing oxidative stress and cellular damage. There is evidence to suggest that carvacrol can enhance the body’s own antioxidant defenses, such as GST and glutathione peroxidase. It may stimulate the expression or activity of these and other antioxidant enzymes, providing a secondary line of defense against oxidative damage [48].

Acetylcholine (ACh) is an essential neurotransmitter but has been associated with energy metabolism and anti-inflammatory effects [12]. Regarding its response to the anti-inflammatory cholinergic system, when not broken down by cholinesterase (ChE), acetylcholine can bind to muscarinic (G protein) or nicotinic (ion channels) receptors of immune cells and act to inhibit the production of pro-inflammatory cytokines. In this way, increased ChE activity reduces bioavailable ACh levels and favors inflammatory processes [56]. In our study, the use of phytogenics may have influenced the fluctuations in serum ChE levels, thereby remodeling the inflammatory response [57]. Initially, a reduction in ChE was observed, followed by an increase. Although these values remained within the reference range for bovines, the reasons for these fluctuations are not yet fully understood.

Immune cells have cholinergic receptors [58,59,60]. This pathway is mediated by ACh, which primarily acts through the vagus nerve and cholinergic receptors on immune cells and peripheral tissues. Lymphocytes, which express ACh and AChE, are responsive to muscarinic and nicotinic receptors [60]. The connection with these receptors leads to the inhibition of the production of pro-inflammatory cytokines, such as tumor necrosis factor alpha (TNF-alpha), interleukins (IL-1, IL-6), and other inflammatory biomarkers [12]. Thus, cholinergic agonists act by inhibiting the synthesis of cytokines, a way of protecting the organism from cytokine-mediated diseases. Thus, the neural signals transmitted by the vagus nerve end up inhibiting the release of cytokines through a mechanism that requires the α7 nicotinic acetylcholine receptor (α7nAChR) [61,62]. Therefore, with a decrease in the hydrolytic activity of AChE, there is consequently an increase in the availability of free ACh to bind to lymphocytes and inhibit inflammation [12,63,64]. However, our results show a higher hydrolytic activity of AChE in cows fed the phytogenic lymphocytes only on day 21, indicating a reduction in the anti-inflammatory activity of ACh specifically on that day. Considering that serum cholinesterase levels were reduced in our study on days 28 and 35, this suggests an increase in ACh bioavailability. Carvacrol has been shown to inhibit the activity of acetylcholinesterase. By inhibiting AChE, carvacrol potentially increases acetylcholine levels in the synaptic cleft. Elevated acetylcholine can activate cholinergic pathways, which are known to exert anti-inflammatory effects [41,45].

Purines within cells play crucial roles in regulating energy metabolism and other cellular processes, while extracellular purines signal through specific purinergic receptors [7,8]. Purines and their derivatives, exemplified by adenosine 5′-triphosphate (ATP) and adenosine, also act as signaling molecules, transmitting signals between cells in various physiological processes, including effects on neurotransmission and the regulation of immune function [65]. The reduction in ADA enzymatic activity in this study reflects an increase in the bioavailability of adenosine, a molecule with known anti-inflammatory effects, capable of reducing the production of pro-inflammatory cytokines [7].

The nucleotides adenosine 5′-triphosphate (ATP), adenosine diphosphate (ADP), and adenosine monophosphate (AMP) are degraded by various nucleotidase enzymes, the product of these reactions being adenosine [66]. This is because NTPDase or CD39 can convert ATP or ADP to AMP by reducing the phosphate load, while ecto-5′-nucleotidase (CD73) further converts AMP to adenosine [67]. The literature has drawn attention to the abnormal metabolism of extracellular ATP (eATP), which has pro-inflammatory effects and promotes several effects on cells, such as inflammation and tissue damage [68]. In this context, the impact of the phytogenic additive was reflected in the increase in NTPDase activity on ATP and ADP substrates, which may be indicative of the decrease in the inflammatory response, as well as increased 5′-nucleotidase activity on AMP substrates [69]. Although adenosine was degraded to inosine due to increased adenosine deaminase activity in our study with a phytogenic additive, inosine, similar to adenosine, exhibits a wide range of anti-inflammatory and immunomodulatory properties [70,71]. These findings suggest that essential oils interfere with cellular and extracellular signaling pathways, although the mechanisms of action are not yet fully understood. Oral administration of curcumin effectively prevented histological damage and changes in NTPDase and AChE activity in the lungs and lymphocytes. This result can be attributed to carvacrol, the main component of oregano essential oil. Use of natural monoterpenes, like carvacrol and thymol, was able to improve the enzyme NTPDase for hydrolyses of ATP and ADP, revealing that these components can be alternatives to improve immune through regulative effects on the purinergic system [72].

The generation and maintenance of cellular energy also rely on the enzymatic action of CK, PK, and AK, which are responsible for efficiently transferring high-energy phosphates and cellular communication that maintains energy homeostasis [73]. CK catalyzes the reversible conversion of phosphocreatine and ADP into creatine and ATP, acting as an energy buffer and allowing rapid ATP regeneration [74]. PK produces ATP from ADP by breaking down glucose to generate ATP [56]. AK catalyzes the interconversion of adenylates, converting two ADP molecules into one ATP and one AMP molecule, and is responsible for cellular energy balance, especially under stress conditions [75]. Our study observed reduced CK and PK activity, possibly related to reduced ATP synthesis and availability [76]. From an inflammatory perspective, this effect could be positive as it indicates a reduction in pro-inflammatory ATP bioavailability [68] due to using phytogenic with the aforementioned anti-inflammatory effects. However, the decrease in ATP could impair cellular homeostasis due to a lack of energy. As a compensatory attempt, the increase in AK may indicate the activation of alternative phosphorylation pathways for ATP production [77]. It is important to note that there are no reports on the effects of phytogenics on energy expenditure regulation in dairy cows. Thus, phytogenics prevented the inhibition of AK activity only. Reports suggest these enzymes are highly susceptible to inactivation by free radicals and oxidation of thiol (SH) groups [78,79], which was not observed in our results.

Given that the cows were at the end of the lactation period, it was only possible to observe the maintenance and persistence of lactation at the end of the experimental period. Additionally, the animals were approximately five months pregnant, and with fetal development, there is an increased energy requirement with adjustments in nutrient partitioning [80]. Despite encouraging health results, no effects of phytogenics on feed intake, milk production, and feed efficiency were observed, as reported when using the same additive at another time [5].

## 5. Conclusions

The cows that ingested the phytogenic blend based on carvacrol, eugenol, cinnamaldehyde, and capsaicin resulted in anti-inflammatory, antioxidant, and immunomodulatory effects. Therefore, they may be a promising alternative to reducing metabolic stress during lactation. Furthermore, there are indications that phytogenics influence immune cholinergic and purinergic signaling pathways, although their effects on energetic metabolism are related to ATP production. The metabolic effects observed in the overall health of Jersey cows fed phytogenics did not negatively impact productive performance. In practice, including phytogenic additives in the cows’ diet benefits the cow’s health in the final third of gestation, when the animal already has a reduced immune response due to advanced gestation. Phytogenics in dairy farming have been used as a performance enhancer, replacing antibiotics, but in this lactation phase, it did not enhance production.

## Figures and Tables

**Table 1:** Chemical composition of experimental diets fed to Jersey cows during the study.

Variables, %	TMR: Supplied in Individual Feeders	* TMR: Provided in Intergado^®^	Pelleted Feed for Robot
Dry matter	38.2	33.7	86.7
Crude protein	19.3	14.4	20.8
Ether extract	3.01	2.67	2.77
Ash	7.08	6.62	5.93
Neutral detergent fiber (NDF)	42.2	51.2	27.6
Acid detergent fiber (ADF)	16.6	22.7	8.98
Gross energy (Kcal)	271	282	379

Note: total mixed ration—TMR (concentrate + silage + hay). The concentrate was formulated based on ground corn (38%), soybean meal (32%), dried distillers grains with solubles (16%), soybean hulls (4.5%), wheat bran (4.5%), mineral premix (3.3%), mycotoxin adsorbent (0.4%), and buffer (1.3%). The mineral core contained calcium (231.40 to 250 g/kg), phosphorus (40 g/kg), sulfur (20 g/kg), magnesium (25 g/kg), potassium (10 g/kg), sodium (70 g/kg), cobalt (15 mg/kg), copper (750 mg/kg), chromium (20 mg/kg), iodine (40 mg/kg), manganese (2000.00 mg/kg), selenium (22 mg/kg), zinc (2850.00 mg/kg), vitamin A (350,000.00 IU/kg), vitamin D3 (100,000.00 IU/kg), and vitamin E (2000.00 IU/kg). * Intergado^®^—smart feeders capable of quantifying how much feed was consumed by the cow during the day but used in this experiment to feed the cows at night.

**Table 2:** Hemograms and serum biochemistry in the blood of Jersey cows fed the control diet, and the diet was supplemented with the phytogenic diet.

Variables	Control	Phytogenic	SEM	P: Treat	P: Treat × Day
Hemogram					
Leukocytes (×10^3^ µL)				0.58	0.05
d1	5.62	5.58	0.15		
d7	5.89 ^a^	5.03 ^b^	0.16		
d14	5.76 ^a^	4.87 ^b^	0.15		
d21	5.56	5.09	0.15		
d28	5.62 ^a^	4.71 ^b^	0.15		
d35	5.61	5.21	0.14		
d42	5.48	5.33	0.14		
Lymphocytes (×10^3^ µL)				0.41	0.03
d1	3.15	3.01	0.11		
d7	3.25 ^a^	2.67 ^b^	0.11		
d14	3.43 ^a^	2.83 ^b^	0.10		
d21	3.25	3.91	0.12		
d28	3.37	3.89	0.12		
d35	3.35 ^a^	2.74 ^b^	0.10		
d42	3.12	3.05	0.11		
Granulocytes (×10^3^ µL)	1.34	1.40	0.13	0.84	0.91
Monocytes (×10^3^ µL)	0.94	0.87	0.26	0.82	0.63
Erythrocytes (×10^6^ µL)	5.32	5.33	0.03	0.95	0.97
Hemoglobin	10.0	9.95	0.09	0.93	0.95
Hematocrit, %	26.9	27.2	0.45	0.92	0.87
Platelets (×10^3^ µL)	302	264	14.6	0.32	0.15
Serum biochemistry					
Total protein (g/dL)	7.57	7.62	0.11	0.76	0.81
Globulin (g/dL)	4.05 ^b^	4.30 ^a^	0.10	0.07	0.11
Albumin (g/dL)	3.51	3.32	0.08	0.65	0.52
Glucose (mg/dL)	43.6	46.7	0.54	0.34	0.28
Cholesterol (mg/dL)	160	147	4.89	0.12	0.21
Triglycerides (mg/dL)	8.80	10.1	0.59	0.64	0.72
Urea (mg/dL)				0.16	0.05
d1	35.6	39.7	2.13		
d7	36.7	37.4	2.09		
d14	45.1	47.3	2.06		
d21	48.6	44.3	2.07		
d28	51.8 ^a^	41.7 ^b^	2.01		
d35	48.3	42.6	2.04		
d42	42.6	38.4	2.01		
GGT (U/L)	14.6	17.2	1.25	0.41	0.57
AST (U/L)				0.02	0.01
d1	91.8	89.7	4.65		
d7	88.3	90.3	5.03		
d14	96.2	84.7	5.01		
d21	92.1	76.1	5.21		
d28	123 ^a^	91.4 ^b^	6.14		
d35	93.7 ^a^	71.2 ^b^	5.85		
d42	80.3	67.3	5.12		
Cholinesterase (×10 U/L)				0.67	0.01
d1	114	118	3.52		
d7	121	124	3.45		
d14	132	128	3.51		
d21	117	102	3.42		
d28	115 ^a^	84.9 ^b^	3.49		
d35	138 ^a^	122 ^b^	3.45		
d42	113 ^b^	137 ^a^	3.52		
BHB (mmol/L)	1.18	1.15	0.03	0.95	0.93

Note: Different letters on the same line show differences between groups (*p* ≤ 0.05).

**Table 3:** Effects of phytogenic addition to the diet of Jersey cows on levels of cytokines (IL-1β, IL-6, and IL-10) and biomarkers of oxidative status.

Variables	Control	Phytogenic	SEM ^2^	Treat ^1^	Treat × Day ^1^
CytokinesInterleukin 1β (pg/mL)	26.9 ^a^	17.8 ^b^	1.68	0.01	0.01
d14	28.3 ^a^	19.4 ^b^	1.71		
d28	27.0 ^a^	16.9 ^b^	1.70		
d42	25.4 ^a^	17.3 ^b^	1.70		
Interleukin 6 (pg/mL)	17.5 ^a^	10.2 ^b^	0.91	0.02	0.01
d14	18.2 ^a^	10.2 ^b^	0.90		
d28	17.9 ^a^	9.98 ^b^	0.93		
d42	16.5 ^a^	10.4 ^b^	0.95		
Interleukin 10 (pg/mL)	36.5 ^b^	48.9 ^a^	3.31	0.01	0.01
d14	35.7 ^b^	41.6 ^a^	3.34		
d28	36.4 ^b^	48.9 ^a^	3.30		
d42	37.6 ^b^	56.4 ^a^	3.32		
Oxidative statusTBARS (nmol/mL)	12.4 ^a^	9.74 ^b^	1.25	0.05	0.12
ROS (% of fluorescence intensity)				0.04	0.01
d14	89.1	87.2	2.35		
d28	83.4 ^a^	69.7 ^b^	6.04		
d42	81.0 ^a^	62.2 ^b^	4.50		
PSH (µmol/L)	3.52	3.68	0.56	0.85	0.72
NPSH (µmol/L)	0.82 ^b^	0.91 ^a^	0.06	0.01	0.01
d14	0.85 ^b^	0.97 ^a^	0.05		
d28	0.82 ^b^	0.99 ^a^	0.05		
d42	0.86 ^b^	1.05 ^a^	0.06		
GST (U GST/mg protein)				0.01	0.01
d14	249	247	7.44		
d28	241 ^b^	222 ^a^	7.89		
d42	210 ^b^	246 ^a^	9.85		

^1^ Within a row, when there are different letters (^a,b^), they differ (*p* ≤ 0.05) or tend to differ (*p* ≤ 0.10) by the t-test. ^2^ Standard error of the mean.

**Table 4:** Performance and milk composition from Jersey cows fed phytogenic or not.

Variables	Control	Phytogenic	SEM	P: Treat ^1^	P: Treat × Day ^1^
Milk production (kg/d)	15.6	15.2	0.30	0.92	0.95
FCM-4% (kg/day) ^2^	17.3	16.2	0.26	0.35	NE
ECM (lbs)	212	199	3.29	0.12	NE
Feed intake, kg DM	14.0	13.8	0.18	0.94	0.89
Feed efficiency	1.11	1.10	0.05	0.97	NE
Milk fat, %	4.75	4.45	0.06	0.45	0.29
Milk protein, %	3.89	3.67	0.04	0.52	0.44
Milk lactose, %	4.64	4.62	0.02	0.97	0.94
Milk total solids, %	14.3	13.7	0.36	0.50	0.43
SCC (×10^3^/mL)	169	229	20.4	0.62	0.49
Body score	3.37	3.41	0.03	0.38	0.73

Note ^1^: There was no effect of treatments or treatment × day interaction (*p* > 0.05). Note ^2^: FCM-4%: 4% Fat-Corrected Milk. Energy Corrected Milk (ECM); NE (not evaluated).

## Data Availability

The data are contained within this article.
